# Collaboration of Japanese Kampo Medicine and Modern Biomedicine 2015

**DOI:** 10.1155/2015/145721

**Published:** 2015-10-08

**Authors:** Kenji Watanabe, Gregory A. Plotnikoff, Takeshi Sakiyama, Heidrun Reissenweber-Hewel

**Affiliations:** ^1^Faculty of Environment and Information Study, Keio University, Fujisawa, Kanagawa 252-0882, Japan; ^2^Penny George Institute for Health and Healing, Allina Health, Minneapolis, MN 55407, USA; ^3^St Marianna University School of medicine, Sugao, Kawasaki, Kanagawa 216-0015, Japan; ^4^Private Clinic for Japanese Medicine and Competence Centre for Complementary Medicine, Technical University of Munich, 82166 Gräfelfing, Germany

The interest expressed by both patients and physicians in integrating logical options from multiple perspectives continues to grow. Even in the most pharmaceutically oriented cultures, patients and physicians recognize that there may be much to learn from the world's many healing traditions. In fact, in Japan, 90% of conventional physicians practice a very unique model that integrates traditional medicine and modern biomedicine. To better understand the scope and the perspectives of this integration, this special issue features several examples on how to apply Kampo medicine in the context of modern biomedicine.

Y.-C. P. Arai et al. showed the effect of the Kampo prescription* kamishoyosan*, which is most often used for the treatment of menopausal syndrome, on pain conditions in patients with intractable persistent dentoalveolar pain (PDAP) disorder. In this devastating condition, no effective conventional treatment has yet been established. However, these authors report that* kamishoyosan* significantly improved the pain intensity in 14 out of 15 PDAP patients.

S. Uehara et al. emphasized the effect of oral administration of* ninjinto* on pediatric chronic intestinal failure which is very difficult to treat by conventional biomedicine. The targeted symptoms were abdominal distension in four patients, diarrhea in three patients, and frequent hospitalization due to infections in two patients. Six out of 7 patients showed an improvement of symptoms. In all four patients with remarkably dilated bowel loops due to chronic intestinal pseudo-obstruction, treatment with* ninjinto* resulted in significant improvement in abdominal X-ray findings.

H. Yasunaga analyzed the reoperation rate after burr-hole surgery for chronic subdural hematoma (CSDH). He analyzed 36,020 patients with CSDH from the national Japanese inpatient database. Among them, 3,889 took the Kampo medicine* goreisan*. Propensity-matched analysis revealed that goreisan use was significantly associated with a lower reoperation rate (4.8%) compared with nonuse (6.2%) and suggested that* goreisan* use reduced the need for reoperation after burr-hole surgery for CSDH. This result is very important in terms of the reduction of medical costs, since many developed countries are facing an increase of medical costs in aged society.

K. Sekiguchi et al. contributed to revealing the mechanisms of action of Kampo medicine in a basic research model. Dermatitis was induced in rat ears by intradermal injection of the bacteria* P. acnes*. A representative Kampo formula for dermatitis,* jumihaidokuto*, was administered orally and compared to the potent anti-inflammatory agent prednisolone (PDN). The authors showed that both JHT and PDN suppressed the extent of dermatitis in this model; however, the mechanism of action was different. Histological examinations revealed that JHT, but not PDN, promoted macrophage accumulation. PDN suppressed the macrophage chemokine MCP-1 while JHT did not affect it. The result supports the clinical belief that Kampo medicines can positively and selectively modulate the host immune system function, while PDN broadly suppresses the host immune system.

One of the unique traditional characteristics in Kampo is the “five viscera” system. T. Tomura et al. developed a five viscera score (FVS) and investigated the validity of this FVS using biological medical data of 50 middle-aged and elderly individuals. The authors concluded that gender, diastolic blood pressure, and HDL-C are related to the liver subscore of the FVS; blood tests for glucose, SGOT, and GGT are related to the spleen subscore; age, BMI, and HSCRP are related to the lung subscore; and HbA1c and creatinine clearance are related to the kidney subscore. This article tries to explain and elucidate the traditional viscera system by modern biomedical markers.

We hope that this wide scope of articles will inspire the readers to consider further the potential benefits from the collaboration of Eastern traditional medicine and Western biomedicine.

